# Neuralgia Treated by Colchicum

**Published:** 1846-11

**Authors:** George Fife


*Neuralgia Treated by Colchicum.* By George Fife, M.D.—Colchicum, of all the means internally employed, deserves most confidence in the treatment of this painful and distressing disease; the advantages of this medicine in these cases seem, if possible, to be greater than those it possesses in gout and rheumatism. From what has been lately said of this medicine, this praise may, by some persons, be regarded as somewhat dubious; such is not, however, to be considered the meaning with which the previous remark is made; on the contrary, I can safely affirm that so far as my experience of this medicine extends, I regard all other means as secondary or auxiliary to it, not only in the disease under consideration, but also in gout and rheumatism, in which it has become fashionable to decry its merits, if not actually to accuse it of prejudicial effects. That such do result from the indiscriminate and irrational manner in which it is employed, cannot be questioned, but in my practice, which now extends over a considerable period, and during which many cases of gout have been treated, I can call to mind no case in which the colchicum has been injurious, or followed by unpleasant effects, which may also be said of its exhibition in rheumatism.

In most of the cases, where local appliances were resorted to, they were used as ointments rubbed over the affected nerve. In some, however, of a more severe character, the morphia was applied to a surface previously blistered. This was, in some cases of sciatica, attended with not only speedy but permanent advantage, and certainly seems highly preferable to Mr. Syme's (of Edinburgh) very farrier-like practice of the cautery.

In cardiac neuralgia the colchicum was especially useful, either with or without a few drops of the tincture of digitalis with each dose, when the action of the heart was much increased as well as irregular. A local application in these cases, of great efficacy in relieving both the inordinate action and intense pain, was the tobacco leaf, slightly moistened and placed over the region of the heart, care always being taken to remove it so soon as any feeling of giddiness, faintness, or sinking, was experienced by the patient. For this simple and truly efficacious application, I am indebted to the late amiable and talented Dr. Macwhirter, of Newcastle.

*Prov. Med. and Surg. Jour.—Braithwaite's Ret.*

